# Are there global shifts in the world science base? Analysing the catching up and falling behind of world regions

**DOI:** 10.1007/s11192-014-1344-1

**Published:** 2014-06-22

**Authors:** Slavo Radosevic, Esin Yoruk

**Affiliations:** SSEES – School of Slavonic and East European Studies, UCL - University College London, 16 Taviton Street, London, WC1H 0BW UK

**Keywords:** Bibliometrics, Absorptive capacity in science base, Static scientific specialization, Dynamic scientific specialization, Revealed comparative advantage, World regions

## Abstract

This paper explores the changing role of world regions (North America, EU15, South EU, Central and Eastern Europe (CEE), Former-USSR, Latin America, Asia Pacific and the Middle East) in science from 1981 to 2011. We use bibliometric data extracted from Thomson Reuter’s National Science Indicators (2011) for 21 broad disciplines, and aggregated the data into the four major science areas: life, fundamental, applied and social sciences. Comparing three sub-periods (1981–1989, 1990–2000 and 2001–2011), we investigate (i) over time changes in descriptive indicators such as publications, citations, and relative impact; (ii) static specialization measured by revealed comparative advantage (RCA) in citations and papers; and (iii) dynamic specialization measured by absolute growth in papers. Descriptive results show a global shift in science largely in quantity (papers) and much less in impact (citations). We argue this should be interpreted as a shift in science’s absorptive capacity but not necessarily a shift of knowledge generation at the world science frontier, which reflects the nature of science systems operating with high inertia and path dependency in areas of their historically inherited advantages and disadvantages. In view of their common historical legacy in science we are particularly interested in the process of convergence/divergence of the catching-up/transition regions with the world frontier regions. We implement an interpretative framework to compare regions in terms of their static and dynamic specialization from 1981–1989 to 2001–2011. Again, our analysis shows that while science systems are mostly characterised by strong inertia and historically inherited (dis)advantages, Asia Pacific, Latin America and CEE show strong catching-up characteristics but largely in the absorptive capacity of science.

## Introduction

Knowledge intensive growth is no longer confined to developed countries (Hollanders and Soete 2010). This is reflected in the increasing importance of scientific knowledge for absorptive capacity of countries. We hypothesize that the science system is being upgraded during the catching up process both in terms of number of outputs, their impact as well as structure. Such upgrading evolves from an ‘absorptive’ to a ‘knowledge frontier generation’ function of science. In catching up economies science systems improve not only in terms of science outputs but also in terms of the disciplinary profile of science output. This profile shifts towards new growing areas of science which represent the knowledge base of new technologies. However, such upgrading is quite a slow and inert process.

In this paper we focus on both static and dynamic scientific capabilities and explore the issues of falling behind and catching up of world regions. We explore long-term changes in world science by differentiating between science as ‘world frontier knowledge activity’ and science as activity which denotes ‘absorptive capacity’. ‘Absorptive capacity’ is defined as “the ability to learn and implement knowledge” (Cohen and Levinthal 1990; Dahlman and Nelson 1995).^1^ In the context of science we define it as the ability to recognize the value of new, external information, assimilate it, and apply it in another context (Cohen and Levinthal 1990). In that respect, science may not generate new world frontier knowledge but it recombines and re-contextualises existing knowledge which thus generates novelty but not necessarily at the world frontier. We explore this dual face of science through changes in world regions’ disciplinary structures. We explore patterns of transformations by distinguishing between changes in absorptive capacity through number of papers and changing participation in world frontier knowledge through impact of papers. We also explore static specialization of regions in major science fields through revealed comparative advantages in publications and citations. We investigate whether science systems operate with high inertia and path-dependency and within their historically inherited advantages and disadvantages or whether static specialization patterns are reactive and prone to significant changes. We explore the nature of such path dependent changes in regions which have undergone major economic changes. This also brings to attention the issue of trade off between excellence and relevance (Radosevic and Lepori 2009)—i.e. whether continuous orientation towards old areas of excellence is the best strategy to also ensure the relevance of science activities to changing technological and industrial knowledge. Finally, we look at the dynamic specialization patterns in major science fields through growth rates of publications over time and compare dynamic specialization patterns with static specialization patterns within an interpretative framework.

We analyse eight world regions within a comparative framework. Apart from the main regions EU15, North America, Latin America, Asia Pacific and the Middle East, we are also interested in South EU, Central and Eastern Europe (CEE) and Former-USSR regions. South EU countries have been going through a major crisis since 2008 after a prolonged period of catching-up both in economy and in science. Former-USSR regions share the communist legacy with the CEE and have been going through a transition process. Previous findings point to a surprisingly strong homogeneity of science systems of the post-communist countries in terms of their disciplinary structure which is explained to a large extent by their common communist legacy (Kozlowski et al. 1999). Hence, we want to explore the extent to which CEE has converged in disciplinary profiles to the EU15, and the extent to which it has diverged from the science systems of the former-USSR. These regions are catching up economies and their technology upgrading has focused largely around improvements in production capability (Kravtsova and Radosevic 2011). R&D has played a role in productivity improvements primarily by facilitating ‘absorptive capacity.’ Their further upgrading will increasingly depend on whether they are building R&D beyond its absorptive capacity as one of the drivers of growth. The same pattern applies to Asia Pacific and Latin America regions. Hence, we are interested in whether it is possible to detect a shift from a largely absorptive function of science knowledge towards a more world knowledge frontier generation in bibliometric data. Asia Pacific and Latin America have been determinedly investing in science over the last few decades (UNESCO 2010). Thus, we explore if there is a ‘global shift’ taking place in science (OECD 2010) between these ‘catching-up’ and ‘core’ regions (North America/EU). In particular, we explore which regions or sub-regions have ‘fallen behind’ and which have been ‘catching-up’ or ‘forging ahead’ (Abramovitz 1986).

Bibliometric analysis is a quantitative method, which analyzes information from the scientific literature database and can provide valuable insights to explain patterns of science and technology (S&T) today and in the future (Martin 1995; Debackere and Glänzel 2004; Sommer 2005; Chuang et al. 2010; Lee et al. 2012). The publication state of scientific literature is also seen as a core indicator for assessing scientific capabilities (Okubo 1997). Therefore, information acquired from bibliometric analysis is very useful and complementary for understanding changes in global S&T, including shifting powers among world regions and countries.

In the next section we explain the dataset and methods of analysis used in this paper along with an interpretative framework to compare regions based on their static and dynamic specializations in the major science areas. “Main findings” Section reports major findings on the comparative positions of world regions in terms of quantity (publications), impact (citations) of bibliometric output and revealed comparative advantage analyses for papers and citations. A comparison of static versus dynamic specialization is also provided under this section in accordance with the suggested interpretative framework. “Conclusions” summarize major results.

## Interpretative framework, data and methods

### Interpretative framework

In general, bibliometric measures are useful tools to investigate the research-based knowledge and thus make it possible to map the structure and changing shape of knowledge resources in the economy and society as a whole. The conventional measures are published research papers in academic journals to represent published output of research activity; citation counts—the number of references to a publication to represent qualified research activity; and the impact measure calculated as the citation counts per paper published. Publication counts refer to the ‘quantity’ of knowledge resources in the economy and society. Whether citation counts refer to the ‘quality’ of knowledge resources is a matter of debate. On specifically this issue, Garfield (1979, p. 361) notes that “What do citation counts measure? While it is theoretically possible that a high citation count could be produced by publishing low-quality work that attracted a lot of criticism, the apparent reluctance of scientists to go to the trouble of refuting inferior work makes such a situation very unlikely.” Conversely, some other scholars are unambiguous in saying “citation counts, that is, the number of references to a publication, cannot tell us about the “quality” of a piece of research…. [they] can only give us an indication of the “impact” research has had on work that follows.” (Katz 1999, p. 2). HEFCE (2009) highlights that the robustness of the bibliometrics varies across the fields of research, lower levels of coverage decreasing the representativeness of the citation information and in areas where publication in journals is the main method of scholarly communication, bibliometrics are more representative of the research undertaken. Therefore, although citation counts are sometimes used as a proxy for ‘quality’ in the bibliometrics literature, they are more appropriately used as a measure of ‘impact.’ In that sense, ‘impact’ measured by ratio of citations to publications has some deficiencies when recent years are included in the analysis. Katz (1999, p. 5) states that generally citations to natural science papers tend to peak in the 3rd to 5th year after publication while in the social sciences they tend to peak in the 5th to 7th year. This means that the recent years are problematic when included in the analysis. For comparison purposes, ‘impact relative to world’^2^ is a more reliable measure than ‘impact’ since it normalizes citation rates according to the world baseline.

The above-mentioned measures and their transformed measures as share of world publications, citations and relative impact (citation impact relative to world), are very useful for descriptive purposes and international comparison. However, they cannot tell us if the country has a relative advantage over others in one specific field of science. In that sense, the ‘Revealed Comparative Advantage’ measure, originally created by Balassa (1965) to show export specialization, is more appropriate. Here we use it to create indices of revealed comparative advantage for published papers (RCAPAP) and revealed comparative advantage for citations (RCACIT). Soete and Wyatt (1983) first introduced it into patent analysis as revealed technological advantage (RTA) index. Since then, the measure has been successfully used in patent analysis to examine specialisation in technology fields (Pavitt and Patel 1988; Meyer 2006; Frietsch and Schmoch 2010; Chen 2011; Zheng et al. 2011) and also in the bibliometrics literature to examine specialisation in scientific fields (Barre 1991 3; Kozlowski et al. 1999; Chuang et al. 2010; Tang and Shapira 2011; Lee et al. 2011, 2012; Harzing and Giroud 2014).

In a bibliometrics context the algebra for the index is set up as follows for citations and published papers (Kozlowski et al. 1999):

1 $$ {\text{RCACIT}}_{j}^{i} = \frac{{\left( {\frac{{{\text{Cit}}_{j}^{i} }}{{{\text{TotCit}}_{j} }}} \right) }}{{\left( {\frac{{{\text{Cit}}_{\text{world}}^{i} }}{{{\text{TotCit}}_{\text{world}} }}} \right)}} $$

where, RCACIT = revealed comparative advantage index based on citations; ${\text{Cit}}_{j}^{i}$ *=* citations in field *i* of country *j*; TotCit_*j*_ = total citations in all fields of country *j*; ${\text{Cit}}_{\text{world}}^{i}$ = world citations in field *i*; TotCit_world_ = world citations in all fields.

2 $$ {\text{RCAPAP}}_{j}^{i} = \frac{{\left( {\frac{{{\text{Pap}}_{j}^{i} }}{{{\text{TotPap}}_{j} }}} \right) }}{{\left( {\frac{{{\text{Pap}}_{\text{world}}^{i} }}{{{\text{TotPap}}_{\text{world}} }}} \right)}} $$

where, RCAPAP = revealed comparative advantage index based on papers; ${\text{Pap}}_{j}^{i}$ = papers in field *i* of country *j*; TotPap_*j*_ = total papers in all fields of country *j*; ${\text{Pap}}_{\text{world}}^{i}$ = world papers in field *i*; TotPap_world_ = world papers in all fields.

The RCA index thus allows for a comparison of regional/national scientific specializations across different scientific fields. When RCA equals 1 for a given scientific field in a given region/country, the percentage share of that field is identical with the world average. When RCA is above 1 the region/country is said to be specialised in that scientific field and vice versa where RCA is below 1. From a methodological point of view, the RCA index was originally formulated to compare relative specialisation in different sectors nation-wise and to allow comparison of the dominance of different sectors of a given nation within a larger group of countries. It should be remembered that these indices (RCA, RTA etc.) are indicators of relative structures and an indicator for ‘international competitiveness’ (Dalum et al. 1996, p. 7). We suggest here that revealed comparative advantage indicates relative scientific performance of individual regions/countries with regard to their scientific publications and citations. Such performance of individual fields of science in a particular region/country can be evaluated by comparing the relative shares of a region/country within the world’s output of scientific publications/citations in individual fields of science and by analyzing changes over time in these shares. For comparability of different regions/countries, these figures need to be normalized by total figures of scientific publications in the region/country and in the world.

However, by using RCA we assume that the scientific frontier moves equally across all S&T areas and that what matters is specialization which reflects countries’ or world regions’ internal science capabilities. However, specialization in growing or stagnant science areas may have different effects on S&T activities. Specialization in growing science areas generates dynamism which stems from increasing S&T opportunities and greater commercialization or implementation potential. From the RCA perspective this may lead to reduced specialization but could be the better option over time compared to strong specialization in stagnant areas. RCA based specialization gives us a static picture and ignores the direction of science changes and differences in growth potential among diverse science areas. So the picture of static (Ricardian) specialization should be complemented by the dynamic (Smithian) specialization (Meoqui 2010).

If we ignore differences in technological opportunities among various scientific areas we cannot properly interpret whether the comparison between two periods in terms of RCA is favourable or unfavourable (whether it is dynamically efficient or not). In other words, we do not have the criteria to assess whether a certain type of specialization enables countries to embark on areas with technological opportunities. This is very important due to changes in technological trajectories and paradigms (Perez 2010) whereby specialization in newly emerging areas may enable higher growth trajectory when compared to specialization in old S&T areas. This enables us to assess the ‘windows of opportunities’ which come from changing technological trajectories and paradigms and whether in retrospect such windows have been captured by catching-up economies (Perez and Soete 1988).

In terms of static efficiency allocation criteria, countries (especially small countries like the CEECs) would be advised to specialize to realize economies of scale and spillovers. However, this view ignores the changing dynamics of science areas and hence specific specialization can be assessed only in view of the changing dynamics of S&T. So what matters is not only relative advantage but also *absolute advantage* or capacity to absorb and generate knowledge in *new areas* irrespective of relative specialization. What matters is the capacity to absorb knowledge that is generated in dynamic areas of science frontier rather than the capacity to generate new knowledge in stagnant areas of science.

**Graph 1:**
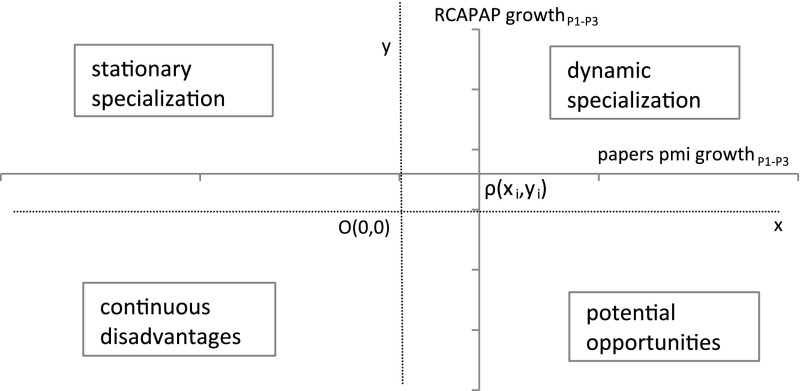
Interpretative framework for RCA growth in papers vs. absolute growth in papers per million inhabitants (pmi) from1981–1989 period (P1) to 2001–2011 period (P3). *Source* Adapted from Molero and García (2008) and Kropacheva and Molero (2013)

Presumably these complementarities are easier to realize in larger S&T systems than in smaller countries/regions. In large economies relative specialization may be lower than in smaller economies. However, in both cases relative specializations cannot be properly assessed beyond absolute advantages or the capacity of regions to embark on high growth areas with S&T opportunities. Therefore, in addition to static (RCA based) specialization we also explore the dynamic aspects of scientific specialization. We assume that absolute and comparative advantages are not necessarily mutually exclusive (Meoqui 2010). We compare publication growth rates of science areas in the world to their growth rates within the region. We also compare growth in RCA indexes of each science field of the world regions with the absolute growth of publications of the field in the world. Then we use both comparisons and merge them in an interpretative framework adopted from the taxonomy in Molero and García (2008) and Kropacheva and Molero (2013). This combines growth in RCA indexes with the growth of publications in the science field from 1981 to 1989 (Period 1) to 2001–2011 (Period 3) (see Graph 1).

We do not use x and y axes which would intersect with each other at O (0,0) for the interpretative framework. However, the coordinates of the intersection point ρ(x_i_,y_i_) of the RCAPAP growth axis and papers pmi growth axis in Graph 1 (as relative to O (0,0) are important for its interpretation. They represent world averages for the specific science areas. For instance, the cut-off point on papers pmi growth axis (x_i_) represents the world average for growth in papers per million inhabitants from Period 1 to Period 3. Likewise, the cut-off point on RCAPAP growth axis (y_i_) represents average growth in RCAPAP for the studied regions. Therefore, the upper right quadrant of the graph denotes growth in the specific science area in terms of number of papers published above the world average as threshold. An above world average growth of papers in specific disciplines coupled with an above world average RCAPAP growth rate from the 1981–1989 period to the 2001–2011 period indicates ‘dynamic specialization’ or specialization in growing areas of world science. An above world average RCAPAP growth in stationary or declining science areas coupled with a below world average growth of papers leads to ‘stationary specialization’ (upper left quadrant). Below world average RCAPAP growth, accompanied with a below world average growth of papers in static or declining science areas, represent areas of ‘continuous disadvantages’ (lower left quadrant). Finally, an above world average growth of papers in growing areas of science but a below world average RCAPAP growth represent ‘potential opportunity’ for increased specialization in fast growing areas (lower right quadrant).

### Data

We extracted data from National Science Indicators (Thomson Reuters 2011), Standard Edition. This is a database of summary publications and citation statistics taken from over 10,000 peer-reviewed journals indexed by ISI during the years 1981–2011. The database covers 180 countries and geographical/political regions of Asia Pacific, European Union (separately for EU15 and EU27), Nordic (Scandinavia), Latin America, the Middle East and OECD. The dataset contains information on fields in the sciences, social sciences, and arts and humanities. The database is available in two versions: a Standard dataset with 21 broad fields in the sciences and social sciences, and a Deluxe dataset of 249 narrower fields in the Sciences, Social Sciences, and Arts and Humanities corresponding to Thomson Reuters’s *Web of Science* ^®^ (*WoS*) categories. In the database, Thomson Reuters counts articles, notes, and reviews as found in Thomson Reuters-covered journals, and omits other types of items and journal marginalia such as editorials, letters, corrections, and abstracts. The country designation reflects the country of the publishing authors. A paper is attributed to all authors’ addresses. For multiple authors from different countries, each country gets full credit for the paper (in terms of overall paper statistics) and citations. This method of counting is appropriate for the purposes of the present study as each paper is an addition to the country’s absorptive capacity being held by the author(s) from each country(s).

### Regions

We selected Asia Pacific, European Union (EU15), Latin America and the Middle East from the Thomson database. In addition to these regions we formed data for CEE, North America, Former-USSR and South EU. Table 1 shows the list of countries included in each group.

**Table 1:** World regions studied in this research

CEE	Bulgaria, Croatia, Czech Republic, Estonia, Hungary, Latvia, Lithuania, Poland, Romania, Slovakia, Slovenia + Czechoslovakia and Yugoslavia
EU15	Austria, Belgium, Denmark, Finland, France, Germany, Greece, Ireland, Italy, Luxembourg, Netherlands, Portugal, Spain, Sweden, UK
EU27	EU15 plus Bulgaria, Croatia, Czech Republic, Estonia, Hungary, Latvia, Lithuania, Poland, Romania, Slovakia, Slovenia
South EU	Cyprus, Greece, Italy, Malta, Spain, Portugal
Former-USSR (excl. EU members)	Armenia, Azerbaijan, Belarus, Kazakhstan, Kyrgyzstan, Moldova, Russia, Tajikistan, Turkmenistan, Ukraine, Uzbekistan
North America	Canada, USA
Latin America	Argentina, Bahamas, Barbados, Belize, Bolivia, Brazil, Chile, Columbia, Costa Rica, Cuba, Dominican Republic, Ecuador, El Salvador, French Guiana, Grenada, Guadeloupe, Guatemala, Guyana, Haiti, Honduras, Jamaica, Martinique, Mexico, Nicaragua, Panama, Paraguay, Peru, Surinam, Trinidad & Tobago, Uruguay, Venezuela
Asia Pacific	Australia, Bangladesh, Brunei, China, Hong Kong, India, Indonesia, Japan, Laos, Macau, Malaysia, Micronesia, Mongolia, Myanmar, Nepal, New Zealand, Pakistan, Philippines, Singapore, South Korea, Sri Lanka, Taiwan, Thailand, Vietnam
Middle East	Bahrain, Egypt, Iran, Iraq, Israel, Jordan, Kuwait, Lebanon, Oman, Qatar, Saudi Arabia, Syria, Turkey, United Arab Emirates, Yemen

The Thomson Reuters database gives aggregate data only for some of the above regions. Moreover, its intra-regional data are ‘cleaned’ i.e. co-authored papers are attributed only once for papers produced by multiple countries within the region but multiple times inter-regionally. For example, if German and Chinese authors produce a paper collaboratively (inter-region collaboration), this paper would appear once in the EU15 data but also in the Asia Pacific data. According to this approach, each paper is an addition to every region’s absorptive capacity. However, according to Thomson’s technical support a paper from two or more countries within the same region is counted just once to prevent double counting. So a paper produced, for instance, by German and Austrian authors collaboratively (intra-region collaboration) would appear only once in the EU15 data. However, for our self-constructed regions (CEE, South EU, North America and Former-USSR), Thomson does not provide data cleaned for co-authorships. National shares of international collaboration are studied in the literature (Glänzel 2001; Zhou and Glänzel 2010), and this also raises the question of multiple counting for co-authored papers from two or more countries within the same region.^4^

Hence we have estimated the weight of intra-regional collaborations across self-constructed regions. The aim was to get an idea of the possible magnitudes of intra-regional collaborations in the total number of papers across regions and thus of possible bias in our data. Unfortunately, we do not have the data to deduct intra-regional collaboration for the entire 30 year period but we have data for such collaboration for the 2008–2012 period.^5^ This allows us to check for the level of intra-regional co-authorships in our self-constructed regions. After calculating intra-regional publication collaborations for CEE, South EU, North America and former-USSR and deducting the figures from self-constructed data for these regions, we found that the share of intra-regional collaborations changes regional shares as a percent of world publications from 0.15 % for the former-USSR to 1.35 % for North America (see Table 7 in Appendix). Intra-regional collaborations as a share of total regional publications are 10 % for CEE, around 7 % for South EU, 5 % for former-USSR and 4 % for North America. Also, the latest period for which our data apply is significantly more intensive in terms of collaborations when compared to the previous periods. Overall, our robustness analysis suggests that the share of collaborations is of such a magnitude that it does not significantly change regional trends.

### Scientific fields

Thomson provides a Standard dataset with 21 broad fields in the sciences and social sciences. These are Agricultural Sciences, Biology & Biochemistry, Chemistry, Clinical Medicine, Computer Science, Economics & Business, Engineering, Environment/Ecology, Geosciences, Immunology, Materials Science, Mathematics, Microbiology, Molecular Biology & Genetics, Neuroscience & Behaviour, Pharmacology & Toxicology, Physics, Plant & Animal Science, Psychiatry/Psychology, Social Sciences-general, Space Science. We have further grouped these broad fields into four major fields: Social Sciences (Social Sciences-general and Economics & Business), Fundamental Sciences (Chemistry, Geosciences, Mathematics and Physics), Applied Sciences (Computer Science, Engineering, Materials Science and Space Science) and Life Sciences (the remaining fields).

### Periods

We study three periods with a total duration of 30 years: 1981–1989, 1990–2000, 2001–2011. The periods are based on decades but also on the occurrence of significant world events. For example, 1989 is viewed as an important year which witnessed radical changes in the modes of science production in the former communist countries. Changes induced since 1989 have led to faster globalization worldwide. The decade from 1990 to 2000 is a transition decade during which the science systems of the post-communist countries underwent turbulent restructuring which led to their stabilization and growth during the third period. Therefore, we mainly base our comparisons on the first (1981–1989) and third (2001–2011) periods.

### Methods

We present the descriptive analysis of indicators related to publications, citations, relative impact and revealed comparative advantages in papers and citations (RCAPAP and RCACIT). In a 2 × 2 matrix, we analyze RCAPAP growth and absolute growth in publications for major scientific fields in regions compared to world averages.

## Main findings

### World science base by regions: publications, citations and relative impact

Table 2 shows the summary changes in world science during 1981–2011 in terms of publications, citations and impact relative to world. There are several important trends.

**Table 2:** Percentage of world publications, percentage of world citations and relative impact in all fields by regions and time periods

	% of world publications			% of world citations			Impact relative to world		
	1981–1989	1990–2000	2001–2011	1981–89	1990–2000	2001–2011	1981–1989	1990–2000	2001–2011
CEE	3.34	3.28	4.31	1.27	1.72	2.83	0.38	0.52	0.66
EU-27	33.24	37.08	37.22	30.35	36.58	40.33	0.91	0.99	1.08
EU-15	31.26	34.96	34.33	29.66	35.70	38.96	0.95	1.02	1.13
South EU	4.07	6.80	9.40	2.97	6.16	9.53	0.73	0.91	1.01
FORMER USSR	7.17	5.19	3.35	1.21	1.35	1.50	0.17	0.26	0.45
ASIA PACIFIC	13.80	18.75	28.29	10.14	13.71	21.13	0.73	0.73	0.74
LATIN AMERICA	1.48	2.34	4.28	0.84	1.52	2.76	0.57	0.65	0.64
MIDDLE EAST	1.62	2.10	4.06	1.35	1.67	2.66	0.83	0.80	0.66
NORTH AMERICA	43.48	40.90	35.24	61.00	57.26	51.09	1.40	1.40	1.45
OECD	84.51	86.34	80.12	95.82	95.53	91.05	1.13	1.11	1.14

*Source* Thomson Reuter’s 2011 National Indicators_Standard ESI

Data for CEE incorporates data for Czechoslavakia and Yugoslavia as well

In terms of publications, CEE is catching up after falling into a decline in the 1990s reflecting the turbulent transition period. Unlike CEE, during the 1990s the South EU region was in a catching up phase and managed to increase its world share, but then slowed down significantly before the current Euro zone crisis.^6^ While CEE was recovering and catching up during the 2000s the former-USSR science systems continued to fall, indicating serious structural crisis of their R&D systems. Despite economic recovery after 1989 their science systems have continued to decline in terms of relative share of world publications although this decline seems to be slowing down. These trends were taking place in the context of EU15’s relatively unchanged position. The relative stagnation of EU15 could have been deeper if it were not for the South EU region. On the other hand, there has been a remarkable catch up of Asia Pacific which indicates the potential for forging ahead i.e. if these trends continue we may see this region overtaking EU15 and North America in its relative share of publications. EU15 and North America have converged in relative shares. However, in the case of North America this convergence happened due to a decline from a very high relative share of 44 to 35 % in 2001–2011 while the EU15’s relative share increased from 31 % in the 1980s to 35 % in the 1990s followed by stagnation at that level in the 2000s. The signs of global shift in world science are also very strong indications of a catch up of Latin America and the Middle East.

In terms of world share of citations, North America continues to lead while EU15 grows albeit at a moderate pace; South EU has been growing strongly as have the Middle East and Latin America while the remarkable catch up of Asia Pacific in terms of quantity (papers) has not yet manifested itself in citations (impact). Overall, changes in citations also suggest a global shift but one that is much more complex as the rise of publications is not automatically accompanied by a proportional increase in recognition or relevance of papers for impact. This reinforces the distinction between the absorptive versus impact dimensions of science.

Countries where absorptive capacity is a driver of investment in science should be expected to have a much lower quality or impact when compared to countries where science is contributing more to the impact (world frontier) dimension of science. Their science systems are largely geared towards following, not leading, the world frontier and their research is largely locally oriented. In contrast, countries that are extending the world frontier and seeking more impact are expected to have a much bigger share of citations than papers. In that respect, North American science seems to generate much more impact when compared to other regions and its impact seems to have increased together with the EU15 which suggest that these are regions operating at the world knowledge frontier.

From the perspective of the distinction between absorptive capacity and world knowledge frontier dimensions of the science base, it is interesting to observe whether regions that have been catching up in terms of publications have also been catching up in terms of impact. In terms of relative impact, world excellence in science is still located in North America followed by EU15 (with the EU15’s share here largely explained by the EU South). The remarkable rise of Asia Pacific and relatively Latin America in both papers and citations is not accompanied by improvements in relative impact which has remained almost unchanged for the last 30 years. This again reinforces the relevance of the distinction between the absorptive and impact dimensions of science, which suggests that science in these largely catching up economies is still mainly focused on its absorptive role. However, a relatively considerable rise of South EU and CEE in both papers and citations is accompanied by sizeable improvements in relative impact showing signs of convergence with the EU27 and EU15. CEE has recorded a significant increase in impact which is somewhat behind the South EU. It is remarkable that this has been achieved during the transition decade, a period when this region’s actual share of papers declined. A distinctly high gap in terms of lower relative impact of the former-USSR science has been gradually closed which suggests that top science results of post-communist region have become more recognised with the opening up of the region, partly due to substantially increased collaborations (Glänzel 2001; Teodorescu and Andrei 2011). Nevertheless top layers of science in the former-USSR remain isolated and seem to be on average of low relative impact.^7^

**Graph 2:**
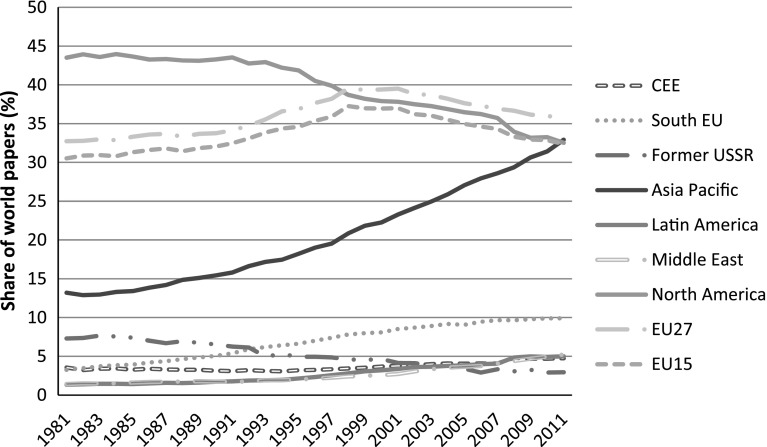
Share of world papers by regions, all fields, 1981–2011

**Graph 3:**
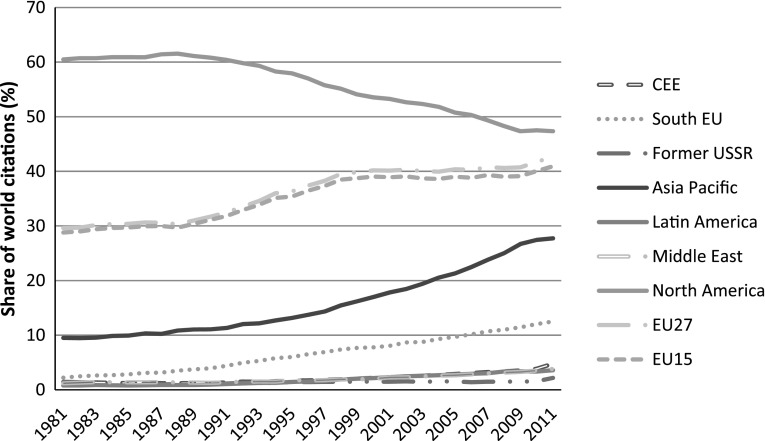
Share of world citations by regions, all fields, 1981–2011

**Graph 4:**
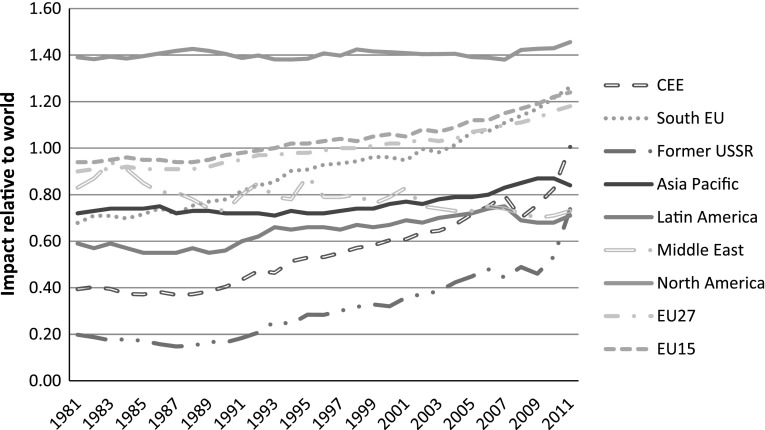
World regions by impact factors relative to world, all fields, 1981–2011

In the rest of this section we present data from Table 2 in graphical form and by years. In this way we visually convey the major trends that are less discernible from Table 2, especially the key ‘turning points’ which are not detectable when compressing data into decades. Graphs 2, 3, 4 are based on the average values of percentage papers published, share of world citations, and relative impact.

Graph 2 shows the US falling behind followed by the recent falling behind of the EU15 from the late 1990s. This ‘global shift’ (OECD 2010) took place due to the rise of Asia Pacific, Middle East, and Latin America regions. However, the increase of the CEE took place in parallel with the continuing decline of the former-USSR. Moreover, stagnating growth dynamics of the South EU region in the years before the Eurozone crisis of 2008 indicate a looming structural crisis of their science systems.

In continuation we demonstrate that this shift in science is more complex and cannot be properly interpreted by only using papers as indicators. It should be interpreted predominantly as a shift in absorptive capacity globally as the science base is a proxy not only for the world knowledge frontier but also for the capacity to absorb external knowledge. In that respect, Asia Pacific (where China, India, Korea and Taiwan produced 45 % of the papers from this region in 2003) is the rising region with South EU trailing at a much lower pace. Moreover, it seems that with the onset of the global financial crisis and Eurozone crisis as its toxic derivative South EU has exhausted its further potential for growth. The Middle East is catching up to the also gradually improving pace of CEE. North America, EU15 and the former-USSR research bases, though at very different levels, have been gradually losing their strength. In that respect, the profile of the global research base has profoundly changed in the last 30 years in favour of newcomers led by Asia Pacific. However, given the dual role of R&D the change is largely a shift in the absorptive role of science, not yet in the share of frontier knowledge generated.

Citations are a proxy for improved impact in science, not mere quantity. Overall, a global shift in citations has been taking place similar to publications: a further decline of North America; slowing down of the EU; stagnation of the former-USSR and a very similar gradual increase of the CEE and Middle East (Graph 3). However, we do not yet see stagnation of South EU in citations which can be expected with some delay. Catching up of Asia Pacific—largely driven by Korea, Taiwan, China and India—is somewhat slower but it seems that it is only a matter of time before quantity in terms of papers get converted into impact growth in terms of citations (Wong and Goh 2012). This positive ‘response effect’ from ‘quantity’ to ‘quality’ may then lead to a fully fledged global shift in science which could change the balance of world regions at the knowledge frontier.

When we plot impact relative to world (Graph 4) we do not observe a global shift which is detected by looking at papers. The absence of any global change is visible through a huge gap in terms of relative impact between North America and the rest of the world. However, relative impact trends of the rest of the world show convergence which is revealing. There seems to be a tendency of EU15 (including South EU) to converge towards North America in terms of relative impact. The remarkable catching up of South EU in terms of relative impact is compatible to trends in citations. Thus, an increased share of South EU in terms of papers has been accompanied by increases in terms of citations and, to an even greater extent, relative impact. In other words, South EU’s improved absorptive capacity of science has been accompanied by its bigger contribution to the world science knowledge frontier. This raises interesting issues about the relations between science, technology and industry knowledge in South EU (Ribeiro et al. 2010). Why have improved absorptive and knowledge generation dimensions of science not translated into long-term economic growth? Are part of the structural problems faced by this part of the EU related to gaps between developed science and absence of demand for applying science in terms of technology and especially industry knowledge? These issues are beyond the scope of this paper; however they are important questions for further research.

There are also convergence processes among the remaining world regions towards a relative impact of 0.8 of the world average. This pattern of convergence is especially strong for the CEECs and the former-USSR, but is absent in the Middle East which actually shows declining relative impact. Asia Pacific has a gradual increase in relative impact although a much smaller increase compared to CEE and former-USSR regions. Former-USSR science systems have declined in terms of relative shares of papers (quantity) but show signs of catching-up in terms of impact. Growth of the CEE in terms of relative impact reflects to a greater extent its growth in relative shares of papers rather than differentiation between quantity and quality as seems to be the case in the former-USSR region. A stagnant relative impact of Asia Pacific and a declining relative impact of the Middle East again show that the global shift is largely a change in absorptive capacity of science, and less a shift in world knowledge frontier activities. If we exclude countries that are leaders in both regions (Japan with 40 % of citations, and Israel with 54.8 % citations in 2003) this may suggest that science in these regions is largely oriented towards absorptive capacities. A majority of these countries are still firmly rooted in the absorptive capacity building stage which underpins their economic growth.

In summary, the global shift in science is largely in terms of quantity (papers) and much less (so far) in relative impact. Hence, the global change is much more about the absorptive capacity of science and much less about regional shifts in the world science frontier. This process is taking place in the context of a gradual shift in terms of quantity of world science towards Asia Pacific and other non-North Atlantic regions, and in the context of a relative decline of the former-USSR science systems in terms of quantity (papers). EU15 trends reveal that South EU is catching up both in terms of quantity and relative impact. After stagnating in the transition decade, the CEE region shows signs of catching up with the EU15 in relative impact but not yet in quantity (papers). These trends may affect the overall EU27 distribution of science knowledge at least in terms of quantity (papers) i.e. in terms of the absorptive capacity of science.

### World disciplinary relative specialization by regions

In this part, we use RCA indexes for papers and citations to explore changes in the relative position of world regions over time. Table 3 shows these trends by four major fields of sciences: life, fundamental, applied and social sciences. Both North America and EU15 present a stable and balanced pattern over time, for RCAPAP and RCACIT ranging around the threshold level 1 for all of the examined major science fields, with the exception of social sciences for EU15 where North America has the sole leadership in specialization during both periods. Asia Pacific’s improvement in both papers and citations in applied sciences, mainly driven by engineering sciences, is noteworthy; whereas Latin America shows a decrease in these measures from 1981–1989 to 2001–2011. In the context of EU, it is notable how South EU was oriented during the 1980s towards applied sciences, of which computer sciences deserves a large share of the credit. Its index of 2.2 for papers is by far the biggest specialization index recorded in all regions. However, this shift towards quantity has not been accompanied by an equal shift towards impact as its RCA for citations has only increased from 1.2 to 1.3 between the two time periods. It seems that the shift towards applied sciences has not been accompanied by a shift in terms of impact which would generate further pull towards the world frontier technological knowledge based on areas around applied sciences (for an example of Italy along these lines, see Daraio and Moed 2011).

**Table 3:** RCACIT and RCAPAP by region, major field and period

	Asia Pacific		CEE		EU15		Former USSR		Latin America		Middle East		North America		South EU	
	1981–1989	2001–2011	1981–1989	2001–2011	1981–1989	2001–2011	1981–1989	2001–2011	1981–1989	2001–2011	1981–1989	2001–2011	1981–1989	2001–2011	1981–1989	2001–2011
RCA-CIT																
Life sciences	1.00	0.85	0.69	0.80	0.97	1.00	0.26	0.36	1.15	1.18	0.89	0.90	1.01	1.05	0.81	0.93
Fundamental sciences	1.22	1.26	1.95	1.61	1.08	1.05	2.44	2.61	1.23	1.18	1.32	1.16	0.93	0.85	1.29	1.15
Applied sciences	1.16	1.33	1.35	1.35	0.97	1.03	1.09	1.59	1.47	1.33	1.31	1.33	1.04	0.94	1.21	1.28
Social sciences	0.32	0.51	0.15	0.31	0.40	0.79	0.06	0.12	0.51	0.47	1.41	0.76	1.44	1.33	0.16	0.46
RCA-PAP																
Life sciences	0.97	0.83	0.84	0.83	1.00	1.03	0.41	0.38	1.20	1.29	0.96	0.88	1.10	1.16	1.04	1.00
Fundamental sciences	1.19	1.16	1.44	1.41	0.96	1.01	1.78	2.24	0.94	0.96	1.06	0.95	0.88	0.84	1.03	1.10
Applied sciences	1.04	1.21	0.96	1.17	0.99	1.04	1.20	1.43	1.01	0.97	1.05	1.00	1.05	0.94	2.22	1.26
Social sciences	0.43	0.53	0.52	0.59	0.61	0.98	0.09	0.21	0.60	0.64	1.15	0.70	1.66	1.48	0.43	0.69

In terms of citations, the CEE has a RCA in fundamental sciences which during the last decade (2001–2011) has been supplemented by stable RCA in applied sciences largely due to a shift to computer and materials sciences (Table 3). A high bias towards fundamental sciences during the 1980s characterised both CEE and former-USSR regions. RCA coefficients for both papers and citations for fundamental sciences in the post-communist world have been by far the highest when compared to other world regions. This has been accompanied by low priority given to life and social sciences. This reflects a belief during communist periods in ‘science as the basis of technological progress’, a belief that was highly skewed towards fundamental sciences. This orientation has remained largely unchanged during the transition period. RCA coefficients remain the highest in this area in both post-communist regions. In former-USSR it actually further increased. So, the post-communist world continues to focus on fundamental sciences when compared to other world regions. This would suggest that these regions face a disproportionally higher problem of the (ir)relevance of its science base for technological and industrial bases. This picture applies equally to both papers and impact which suggest that ‘quantity’ breeds ‘impact.’ Whether continuous orientation towards old areas of excellence is the best strategy to ensure the relevance of science activities to changing technological and industrial knowledge is a matter of debate (Radosevic and Lepori 2009).

In order to systematically explore shifting revealed comparative advantages of world regions we design matrices comparing RCA both in terms of papers and citations in two periods: 1981–1989 and 2001–2011 (Tables 4, 5).

**Table 4:** Changes in revealed comparative advantages of world regions in four major fields of science as shown by the number of papers published (RCAPAP) in 1981–1989 and 2001–2011 periods

	RCAPAP >1 (1981–1989) (old advantages)	RCAPAP <1 (1981–1989) (old disadvantages)
RCAPAP >1 (2001–2011) (new advantages)	*Areas of continuous advantages* CEE: fundamental EU15: life Former-USSR: fundamental, applied North America: life, social South EU: life, fundamental, applied Latin America: life Asia Pacific: fundamental, applied Middle East: applied	*Areas of newly gained advantages* CEE: applied EU15: fundamental, applied
RCAPAP <1 (2001–2011) (new disadvantages)	*Areas of lost advantages* North America: applied Latin America: applied Middle East: fundamental, social	*Areas of continuous disadvantages* CEE: life, social EU15: social Former-USSR: life, social North America: fundamental South EU: social Latin America: fundamental, social Asia Pacific: life, social Middle East: life

**Table 5:** Changes in revealed comparative advantages of world regions in four major fields of science as shown by the number of citations (RCACIT) in 1981–1989 and 2001–2011 periods

	RCACIT >1 (1981–1989) (old advantages)	RCACIT <1 (1981–1989) (old disadvantages)
RCACIT >1 (2001–2011) (new advantages)	*Areas of continuous advantages* CEE: fundamental, applied EU15: fundamental Former-USSR: fundamental, applied North America: life, social South EU: fundamental, applied Latin America: life, fundamental, applied Asia Pacific: fundamental, applied Middle East: fundamental, applied	*Newly gained advantages* EU15: life, applied
RCACIT <1 (2001–2011) (new disadvantages)	*Areas of lost advantages* North America: applied Asia Pacific: life Middle East: social	*Areas of continuous disadvantages* CEE: life, social EU15: social Former-USSR: life, social North America: fundamental South EU: life, social Latin America: social Asia Pacific: social Middle East: life

First, Table 4 shows that regional advantages and disadvantages are quite persistent features of world science. In a 30 year period, only two regions have seen newly gained advantages in terms of RCAPAP. EU15 has gained RCAPAP in fundamental and applied sciences and CEE in applied sciences. This was followed by the loss of RCAPAP by North America and Latin America in applied sciences and the Middle East region’s loss of RCAPAP in fundamental and social sciences. At an aggregate level, science systems operate with high inertia and in the areas of their historically inherited advantages and disadvantages.

Second, Table 5 shows that the persistence of regional advantages and disadvantages is even more pronounced in terms of citations and impact. In a 30 year period only two regions have seen newly gained advantages in terms of RCACIT. Only the EU15 has increased RCACIT in life and applied sciences and only three regions have lost relative advantages. The CEE region’s newly gained relative advantages in applied sciences papers has not yet been followed by RCA in terms of citations. From a global perspective, it is interesting to note North America’s loss in applied sciences and that of Asia Pacific in life sciences. The former-USSR region has not gained new major areas of comparative advantage and remains, at aggregate level, specialized in fundamental and applied sciences and de-specialized in life and social sciences. This suggests that scientific specializations are historically rooted and highly path dependent even in regions which have undergone major changes in terms of economic regime and openness of their science system.

**Fig. 1:**
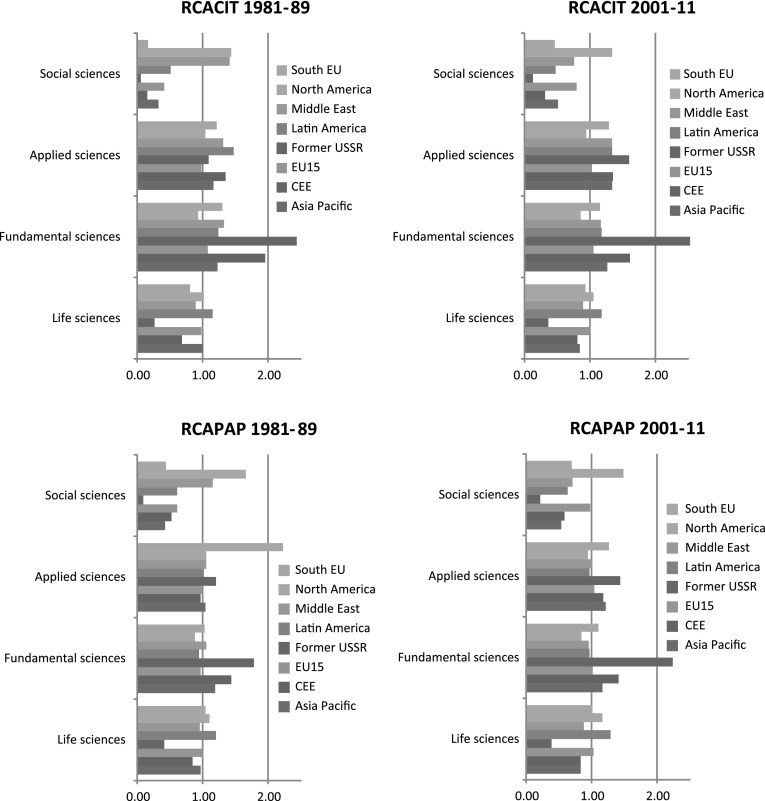
RCA changes in citations and papers across four major science areas by world regions

RCA aggregate data suggest that there have not been significant changes in the disciplinary structures of world regions’ science systems. Despite significant institutional and political changes, science systems in the world operate with relatively high degrees of autonomy which could be explained less by institutional differences and more by the nature of scientific capabilities which are still highly localised, cumulative and path dependent. Thus, a global shift in science has taken place in terms of share of overall papers (the absorptive dimension of science) but not in terms of disciplinary specializations. Finally, amid a strong persistence of disciplinary structures it is significant to note that CEE, a relatively small region, has shifted excessive specialization from RCAPAP in fundamental sciences towards applied sciences. For detailed graphic representations of RCA changes across the two time periods see Fig. 1 in Appendix.

**Graph 5:**
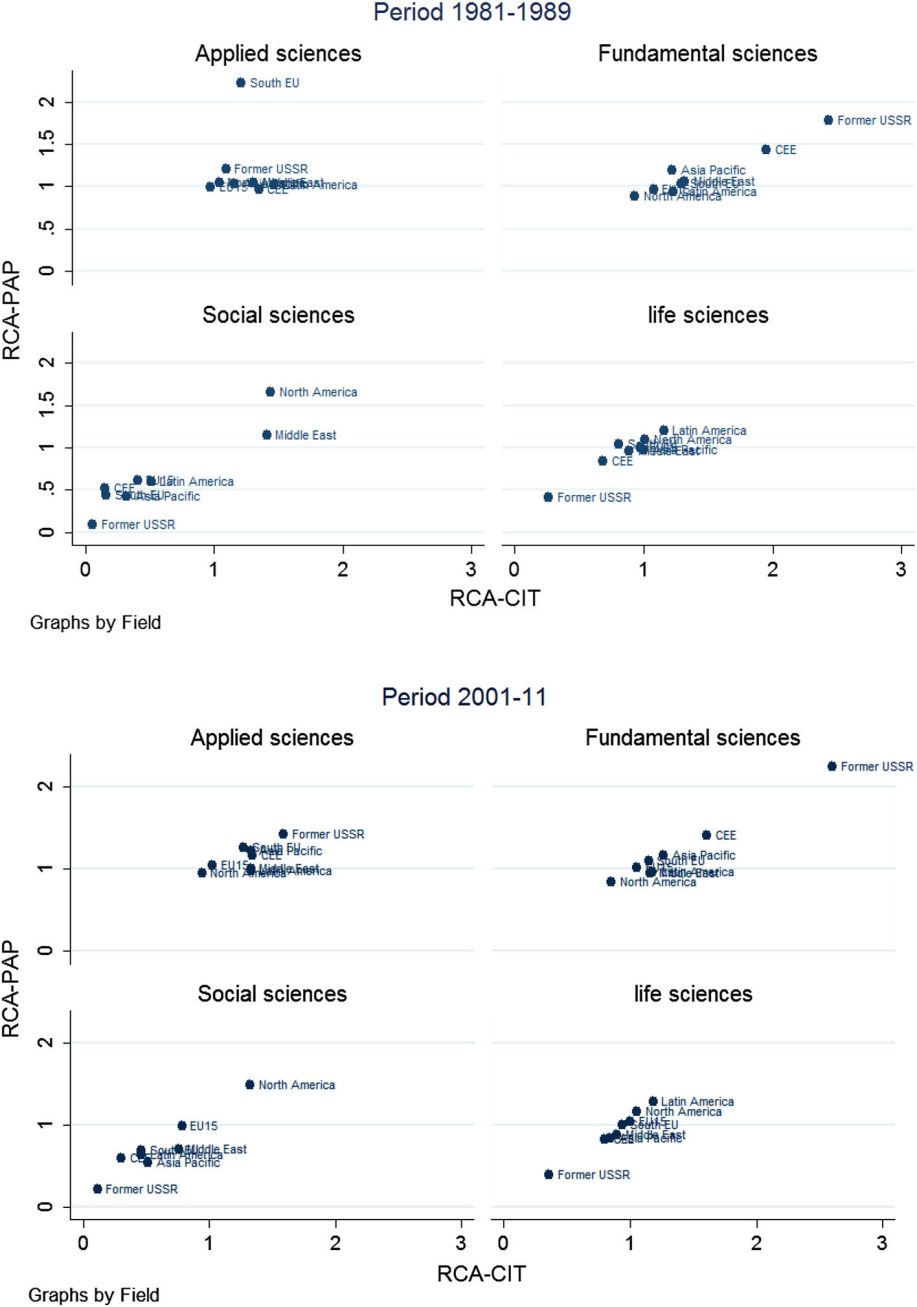
Scatter diagrams for RCAPAP and RCACIT by four major fields of science by world regions and by periods 1981–1989 and 2001–2011

Graph 5 is based on the scatter diagrams of RCACIT and RCAPAP for world regions by four major areas of sciences in two periods. In the 1981–1989 period we can see the strong specialization of CEE and former-USSR in fundamental sciences, strong specialization of South EU in applied sciences, strong specialization of North America and Middle East in social sciences and strong de-specialization of former-USSR in life sciences and in social sciences.

In the 2001–2011 period, there has been a limited shift in RCA among regions (Graph 5). However, a few features remain pronounced. While former-USSR continued to specialize in fundamental sciences and its science system has become extremely unbalanced, it continued to de-specialize in life sciences and social sciences. CEE reduced its excessive specialization in fundamental sciences and shifted more towards applied sciences. On the other hand, South EU’s very strong specialization in applied sciences in the first period led to strong de-specialization in the subsequent period. North America continued to be strongly specialized in social sciences while the Middle East de-specialized in that area. Overall, this would suggest that the EU science specializations have become more homogenous while former-USSR continues to be an outlier in terms of RCA.

### Static and dynamic specialization

In analysing regions’ specializations we have so far ignored differences in S&T opportunities. In terms of static efficiency allocation criteria, countries would be advised to specialize as that is also the way to realize economies of scale and spillovers, especially for small countries. However, this view ignores the changing dynamics of science areas and hence specific specialization can be assessed only in view of the changing dynamics of S&T. So what matters is not only relative advantage but also *absolute advantage* or capacity to absorb and generate knowledge in *new areas* irrespective of relative specialization. The capacity to absorb knowledge generated at dynamic areas of the S&T frontier matters more than the capacity to generate new knowledge in stagnant areas of scientific frontier.

S&T advantages do not necessarily emerge from relative specialization irrespective of science area; rather they arise from a mixture of critical masses in different high growth science areas. Such a combination may create a self-reinforcing process of science based growth through increasing returns based on complementarities among different areas (for example, biophysics), not necessarily based on economies of scale in one specific field. The underlying point is that mechanisms of interaction among science areas are much richer and more complex than assumed from a comparative advantages perspective. For example, a strong specialization in an old science area with limited S&T opportunities may be inferior if not accompanied by entry into the new growing science areas.

Hence in this section we return to the interpretative framework we propose in Graph 1 and investigate the complementarities between the static (RCA based) aspects of specialization and the dynamic aspects of science specialization. We show the results of this analysis based on RCAPAP only, since RCAPAP and RCACIT follow similar patterns in world regions. We do not include social sciences in this analysis as it is clear from the previous analysis that North America is the leading region in this field and all other regions have either lost advantages or lag far behind. Evidence shows that the majority of social science publications are in English where North America and the UK have obvious advantages (Harzing and Giroud 2014).

**Graph 6:**
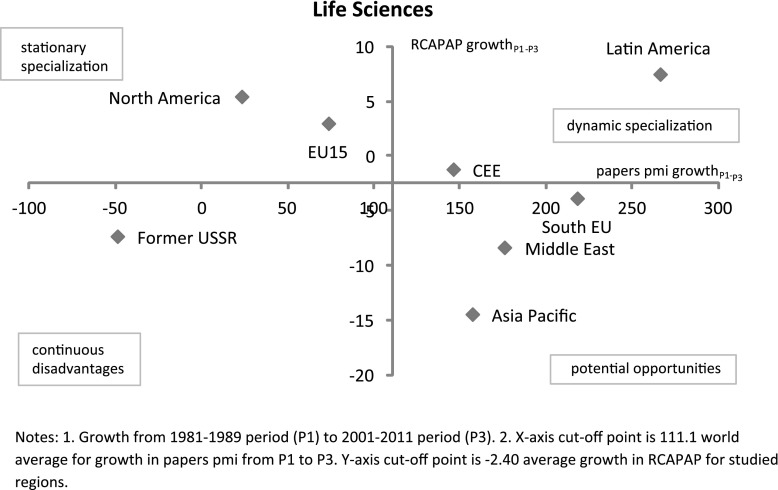
RCAPAP growth vs. absolute growth in papers per million inhabitants in life sciences by world regions from the 1981–1999 period to the 2001–2011 period

Graph 6 compares RCAPAP growth in *life sciences* with growth in papers per million inhabitants from 1981–1989 (period 1) to 2001–2011 (period 3). A static specialization analysis (Graph 5) shows the former-USSR region as an outlier and the other regions as more or less convergent to each other in both periods. When dynamic aspects are considered, life sciences are actually an area of diverging dynamics among the world regions. The world average for growth in published papers from the 1981–1989 period to the 2001–2011 period is 111.1 % and the average growth rate in RCAPAP is −2.4 %. North America continues to dominate in RCA but its slow growth in published papers puts it in the ‘stationary specialization’ quadrant. The EU15 shows a similar pattern and is also below world average for growth in published papers, albeit with a higher growth rate in papers per capita compared to North America. On the other hand, Latin America has significantly increased its relative focus on life sciences and is increasing significantly its number of papers per capita (Krauskope et al. 1986). CEE, albeit lying in the ‘dynamic specialization’ quadrant, does not exhibit a positive growth rate in RCAPAP (indeed it is stagnant at 0.8 in period 1 and 0.8 in period 3) but this is still an above world average growth rate coupled with above average growth rate in papers. Former-USSR continues to lag in life sciences in relative and absolute terms. Asia Pacific has increased the number of papers per capita to a similar extent though it is still de-specialized in life sciences. A similar growth pattern prevails for South EU and the Middle East. South EU has the most favourable position among the three regions in this category with the highest levels of relative and absolute growth.

**Graph 7:**
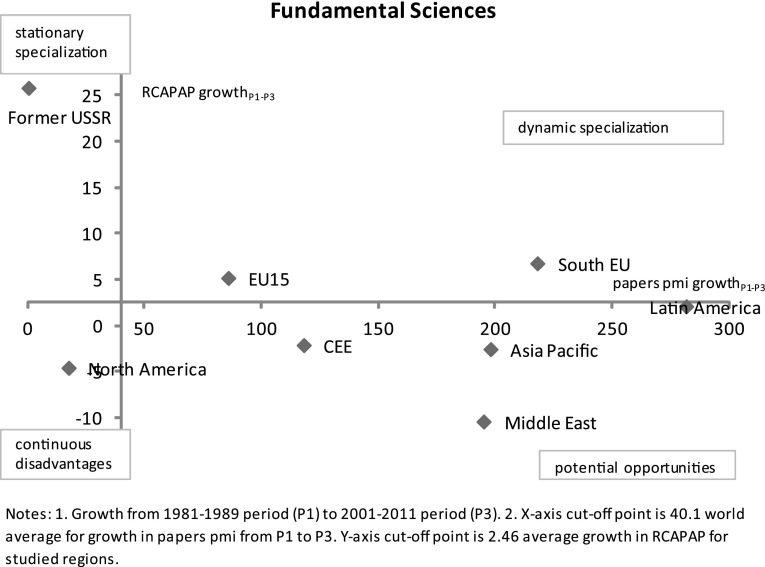
RCAPAP growth vs. absolute growth in papers per million inhabitants in fundamental sciences by world regions from the 1981–1989 period to the 2001–2011 period

Graph 7 compares RCAPAP growth in *fundamental sciences* with growth in papers per million inhabitants from 1981–1989 to 2001–2011 period. It shows the former-USSR as strong outlier in the quadrant of ‘stationary specialization.’ When dynamic aspects are examined, fundamental sciences depict diverging dynamics among the world regions. The world average for growth in published papers between the two periods is 40.1 % and the average growth in RCAPAP is 2.5 %. The former-USSR shows excessive relative specialization in fundamental sciences, an unusual rise of RCA by 25 % although its absolute growth in per capita papers is almost 0 %, below the world average. This may point to a decline from the saturation point reached in the last decade, especially in physics which is traditionally a very strong area in the former-USSR region (Wilson and Markusova 2004). EU15 and South EU appear in the ‘dynamic specialization’ quadrant as they have higher than world average growth rates both for relative (RCA) and absolute (growth of per capita papers) measures. South EU shows a more dynamic pattern than the EU15 for both dimensions. In the light of the recent crisis in South EU region, this raises further questions about the relevance of investment in fundamental science research for applied/engineering areas, including domestic innovation activities. North America is the only region placed in the ‘continuous disadvantages’ quadrant with below world average growth rates in both absolute and relative terms. It shows that over the 30 year period there has been a downward trend in fundamental sciences in North America. Latin America, Asia Pacific, CEE and the Middle East are in the ‘potential opportunities’ quadrant. Among all regions, Latin America has the most favourable position with the highest growth rate in papers.

**Graph 8:**
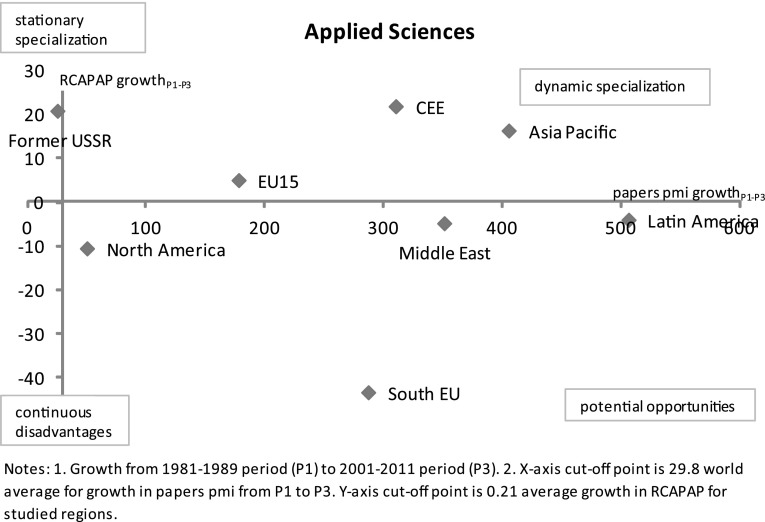
RCAPAP growth vs. absolute growth in papers per million inhabitants in *applied sciences* by regions from the 1981–1989 period to the 2001–2011 period

Lastly, Graph 8 compares RCAPAP growth in *applied sciences* with growth in papers per million inhabitants from the 1981–1989 period to the 2001–2011 period. When we look at dynamic aspects, applied sciences is an area with strong diverging regional dynamics. The world average for growth in published papers between two periods is 29.8 % and the average growth in RCAPAP is 0.2 %. Former-USSR dominates in terms of RCA but is below the world average growth rate in terms of published papers so is characterised as ‘stationary specialization.’ On the other hand, Asia Pacific, CEE and EU15 have all significantly increased their relative focus on applied sciences as well as increased considerably their number of papers per capita. Latin America, the Middle East and North America have increased their number of papers per capita to above world average though their relative specialization is still very low which qualifies them as regions of limited but nevertheless ‘potential opportunities.’ Latin America, among the three regions in this category, has the most favourable position with high rates of growth in papers while South EU has been de-specializing in applied sciences.

In summary, a combined analysis of static and dynamic specialisations shows strong historically rooted regional patterns with only some new developments (Table 6). First, North America and EU15 continue to be specialized in life sciences. This is also the fastest growing science area which by itself moderates changes in the ‘global shift.’ By the same token, the former USSR region continues to be strongly specialized in fundamental and applied sciences. Second, Asia Pacific has been specializing in applied sciences with close links to its manufacturing capabilities. This field is one of unique strength for the Asia Pacific especially given the increasingly large science potential of the region. The EU has also been specializing in both applied and fundamental sciences but not at the same level of both absolute growth and relative specialization. The areas of dynamic specialisation i.e. high absolute growth of publications and increasing relative specialisation have been characteristic of smaller regions. CEE has been specializing in life sciences and applied sciences, Latin America in life sciences and the South EU in fundamental sciences. However, while these shifts have been moderate they are still notable. Third, North America continues to be highly specialized in life sciences while the former USSR continues to be de-specialized in life sciences. These static specializations are moderated by the high growth of publications in life sciences and by significantly lower growth rates in fundamental and applied sciences publications.

**Table 6:** Regions compared by type of relative and dynamic specialization in areas of science from the 1981–1989 period to the 2001–2011 period

	North America	EU15	South EU	CEE	Asia Pacific	Latin America	Middle East	Former-USSR
Life sciences	Stationary specialization	Stationary specialization	Potential opportunities	Dynamic specialization	Potential opportunities	Dynamic specialization	Potential opportunities	Continuous disadvantages
Fundamental sciences	Continuous disadvantages	Dynamic specialization	Dynamic specialization	Potential opportunities	Potential opportunities	Potential opportunities	Potential opportunities	Stationary specialization
Applied sciences	Potential opportunities	Dynamic specialization	Potential opportunities	Dynamic specialization	Dynamic specialization	Potential opportunities	Potential opportunities	Stationary specialization

## Conclusions and discussions

In this paper we explored the changing role of world regions in world science. We used bibliometrics data spanning 30 years and specifically explored changing shifts in papers and citations. In addition to descriptive statistics we used RCA indicators applied to citations and papers. We then complemented the analyses with an investigation of stagnant and dynamic specializations of regions in major science areas within an interpretative framework.

First, we show that there has been a global shift in science but largely in quantity (papers) and much less—or not yet—in impact (citations). The change is characterised by a gradual shift in papers towards the Asia Pacific and other non-North Atlantic regions and by a decline of the former-USSR science systems.

Second, at an aggregate level science systems operate with high inertia and in areas of their historically inherited advantages and disadvantages. Within largely unchanged areas of regional advantages and disadvantages over the past 30 years, only the EU15 has gained RCA in papers in fundamental and applied sciences and CEE in applied sciences. Only the EU15 has gained RCA in citations in life and applied sciences. Other world regions have not gained advantages in new areas: this shows a very strong persistence of world science specialization patterns. De-specializations are more frequent than increased specializations. North America has lost advantages in applied sciences in both papers and citations; while the Middle East has lost advantages in fundamental (papers) and social sciences (both papers and citations), and Asia Pacific has lost advantages in life sciences (citations).

Third, at a more detailed level we highlight three major changes. First, South EU’s loss of excessive specialization in applied sciences. Second, CEE and former-USSR were excessively specialized in fundamental sciences during the communist period. Subsequently, CEE has reduced its specialization in fundamental sciences while the former-USSR continued with its excessive specialization and has further de-specialized in life sciences. Third, we find an excessive specialization of North America and the Middle East in social sciences (albeit for opposite reasons) which was followed by reduced specialization of the Middle East and by continuous high specialization of North America in social sciences.

The former-USSR is unique among the world regions as it has performed below the world average in all areas of science in terms of absolute growth of papers. Thus, it represents a very strong case of ‘falling behind’. It is though excessively above the world average growth rates in RCAPAP for fundamental and applied sciences, which suggests that these science systems are highly unbalanced (Yang et al. 2012). While the former-USSR continues on its divergent path (specifically in fundamental sciences when compared to other regions), the CEE region has been showing signs of convergence with the rest of the world with a stable divergence path from the former-USSR. This demonstrates divergence from the common institutional features shared during the pre-transition period (Radosevic and Auriol 1999; Radosevic 2002).

In this context, CEE, South EU, Asia Pacific, Latin America and Middle East are catching-up regions. In all science areas their dynamic specializations (as expressed in absolute growth rates in papers) are above the world average growth rates and they are even exceeding the rates of world frontier regions (North America and EU15). Among the catching-up regions, there is a sharp difference between South EU and Asia Pacific in terms of the relevance of their science specializations for their industry/technology base. Asia Pacific seems to follow a science policy which prioritizes applied sciences (see for example Wong 2013; Harzing and Giroud 2014) whereas South EU has opted for fundamental sciences. Asia Pacific’s preference for prioritizing engineering sciences could be more conducive to the absorptive function of science than that of South EU’s fundamental sciences preference. Similar issues could be raised in the context of excessive specialization of the former-USSR in fundamental sciences and divergence of the CEE region from this specialization towards applied sciences.

Among the catching-up regions, Latin America’s dynamic position in life sciences is notable. This is the result of favourable science policies in the past few decades, especially in Argentina and Brazil (Garg 2003; Yang et al. 2012; Harzing and Giroud 2014). Latin America has also managed to reach above world average growth rates in published papers in both fundamental and engineering sciences. This region emerges as the second most dynamic region. The Middle East, on the other hand, is characterised by a decline in relative specialization in all science areas but managed to keep its levels of published papers above the world average.

EU15 does show a steady but slow increase in terms of both absolute and relative growth rates as expected from a world frontier region. However, the apparent decline of North American science would need further research. This may be explained by declining R&D funding, fewer immigrant scientists as a result of stricter rules after 9/11, or a reduced role of manufacturing. It is also interesting to note that fundamental sciences have been a continuously disadvantaged area in North America over the course of the last 30 years.

Finally, there seems to be a division of labour in global sciences with North America being strongly specialized in life sciences, Asia Pacific in applied sciences, EU15 in all three areas and former USSR in fundamental sciences. Other regions are strongly affected by their core countries, which in particular strongly influence the entire region’s specializations. Thus CEE is similar to the EU in becoming more specialized in applied and life sciences and moving away from fundamental sciences. Latin America has been moving towards life sciences tied to strong cooperation with the US. The Middle East remains relatively under-specialized, which reflects the very strong role of science in its absorptive capacity in that region.

Finally, our results indicate the need to further explore the relationship between the science base in its absorptive and knowledge frontier function and its relationship to technological and industrial knowledge.

## Appendix

See Table 7 and Fig. 1.

**Table 7:** Estimated share of intra-regional collaborations in % of world publications and % of intra-regional collaborative publications in total regional papers

	Estimated share of intra-regional collaborations in % of world publications				% of intra-regional collaborative publications in region’s total papers			
	North America	South EU	CEE	Former USSR	North America	South EU	CEE	Former USSR
2008	1.35	0.75	0.50	0.15	3.98	7.76	10.28	4.86
2009	1.32	0.73	0.48	0.15	3.98	7.49	10.35	4.48
2010	1.34	0.75	0.49	0.15	4.04	7.54	10.55	5.10
2011	1.26	0.70	0.46	0.14	3.88	7.07	9.75	4.72

Data taken from http://www.ukresearchbase2013.co.uk based on Scopus

## Footnotes

1 For bibliometric analysis of research on ‘relative absorptive capacity’ in different areas see Martinez et al. (2012).

2 ‘Impact’ is citations in a field divided by published papers in that field (C1/P1). ‘Impact relative to world’ is citation impact in a field divided by citation impact for the world (all fields) (C1/P1)/(Cw/Pw). This is a baseline comparison to the world.

3 Barre (1991) named it as Revealed Scientific Advantage and calculated the index for published papers; hence Barre examined only quantity. In this study, we approach the matter both from quantity and impact perspectives and thus distinguish between published papers and citations. We prefer to use the terms Revealed Comparative Advantage for papers (RCAPAP) and for citations (RCACIT) separately.

4 We appreciate anonymous reviewer’s comments regarding this issue.

5 Data are available at http://www.ukresearchbase2013.co.uk. Data cover articles, reviews and conference proceedings. Note that Thomson data do not cover conference proceedings. Thus, the addition of conference proceedings increases data on intra-regional collaborations compared to the Thomson data.

6 This observation is based on annual data which we do not report here.

7 Later on we show that former-USSR is also the region with the most uneven RCA indexes which suggest that pockets of former-USSR science are much more developed than others.
